# Impact of Personal Values on School Engagement Among Chinese Adolescents: Mediating Effects of Achievement Goals

**DOI:** 10.3390/bs15010076

**Published:** 2025-01-16

**Authors:** Tingyu Gu, Xiaosong Gai

**Affiliations:** 1 School of Psychology, Northeast Normal University, 5268 Renmin Street, Changchun 130024, China; guty577@nenu.edu.cn; 2 Research Center of Mental Health Education in Northeast Normal University, Key Research Institute of Humanities and Social Science in Universities in Jilin Province, Northeast Normal University, Changchun 130024, China

**Keywords:** personal values, school engagement, achievement goals, adolescents

## Abstract

Much of past research has centered on the impact of personal values on career progression. Yet, the connection between personal values and academic performance remains under-explored, especially the mechanisms through which they relate. Additionally, the relative strength of the correlation between different types of personal values and academic performance has yet to be examined. This research aimed to examine the effect of various personal values on school engagement among Chinese adolescents, as well as the role of four achievement goals as potential mediators. We surveyed 700 senior high school students from a public school in Changchun, Jilin province, China (M_age_ = 16.91, SD_age_ = 0.43, 55.57% male). Participants completed the Personal Values Scale, Achievement Goal Orientation Scale, and School Engagement Scale. Materialistic values were positively correlated with performance avoidance goals, which in turn were negatively related to school engagement. In contrast, self-improvement values were positively associated with school engagement. This relationship was mediated by a higher number of mastery approach goals and fewer performance avoidance goals. Self-transcendence values were linked to an increase in mastery approach goals, which were in turn positively related to school engagement. Additionally, although both self-improvement and self-transcendence values positively correlated with adolescents’ school engagement, the correlation was stronger for self-improvement values. This study makes a unique contribution by showing that personal values are linked to school engagement through achievement goal orientations, thereby supporting and expanding the future-oriented motivation and self-regulation model. The practical value of this study lies in demonstrating that promoting self-improvement and self-transcendence values, particularly self-improvement values, fosters positive achievement goal orientations, such as mastery approach goals, which in turn enhance school engagement.

## 1. Introduction

Personal values are a core aspect of one’s self, distinct from traits, motives, goals, or attitudes (Hitlin, 2003; Roccas et al., 2002, 2014). They are broad, desirable goals that drive people’s actions, acting as guiding principles in areas like academics and careers (Schwartz, 1992). Schwartz (1994, 2013) identifies ten types of personal values, which are universalism, benevolence, tradition, conformity, security, power, achievement, hedonism, stimulation, and self-direction. These values interact dynamically and are organized along two bipolar dimensions, illustrating conflicts between opposing values. Harris (2011) further refines this understanding, differentiating personal values from goals. While goals are tangible objectives, personal values represent intangible directions we strive towards.

In this domain, much research has concentrated on the role of personal values in career development in college students and employees. This includes their influence on career choices (Añaña & Nique, 2010; Sagiv et al., 2017; Schwartz & Bardi, 2001), career decision-making (Fearon et al., 2018), work engagement (Sato et al., 2021), perceived career success (Gaile et al., 2022), and career efficacy (Francescato et al., 2020). Specifically, personal values play a role in guiding career paths and decisions in college students and employees (Añaña & Nique, 2010; Sagiv et al., 2017; Schwartz & Bardi, 2001). Certain value types are linked to increased work engagement, higher perceived career success, and greater career efficacy (Francescato et al., 2020; Gaile et al., 2022; Sato et al., 2021).

In fact, personal values have already been formed during high school (Johnson, 2001; Johnson & Elder, 2002). For these students, the emphasis is more on academic performance than on immediate career choices. Thus, it is crucial to examine how distinct personal values influence the academic outcomes rather than the career development of high school students. Although several review studies have suggested that personal values can influence learning approaches and student achievements (Gamage et al., 2021; Rouse & Austin, 2002), they did not directly test these relationships. Roeser et al. (2002) conducted empirical research that provided evidence on how specific personal values affect academic performance. They used a cross-sectional design and selected 97 adolescents from two middle schools in the San Francisco Bay area to participate by completing a questionnaire. The results demonstrated that middle school students’ intrinsic values positively influenced their cognitive and behavioral outcomes in learning, such as cognitive engagement. However, these investigations have primarily focused on the effects of specific personal values on academic performance. There remains a significant scope for further exploration, such as determining if different personal values vary in their impact on academic performance.

To bridge this research gap, our study set out to investigate the impact of various personal values on the academic performance of high school students and to explore the mediating mechanisms at play.

### 1.1. Personal Values and School Engagement

Although various metrics, like learning persistence (Brown et al., 2008; Rayle et al., 2006; Tracey & Robbins, 2006) and school engagement (Skinner et al., 2009), can gauge adolescents’ academic performance, this study chose school engagement as its indicator. This decision was based on school engagement’s potential for a more direct and consistent correlation with academic achievement (Skinner et al., 2009). School engagement is characterized as a consistent positive psychological state associated with learning (Schaufeli et al., 2002). This multifaceted concept encompasses cognitive, emotional, and behavioral aspects (Fredricks et al., 2004; Jimerson et al., 2003). Cognitive engagement involves strategic or self-regulated learning (Pintrich & De Groot, 1990). Emotional engagement encompasses a sense of belonging to a school, enjoying school learning, and valuing academic success (Voelkl, 1997). Behavioral engagement refers to active participation and involvement in academic activities (Fredricks et al., 2004). These dimensions can be combined into a comprehensive score representing school engagement.

Several studies have examined the impact of personal values on adolescents’ school engagement (Roeser et al., 2002; Rouse & Austin, 2002). Specifically, research has shown that intrinsic values, which emphasize learning potential and creativity (Johnson, 2001; Johnson & Elder, 2002), correlate positively with students’ engagement (Roeser et al., 2002). This observation holds true across diverse racial backgrounds and socio-economic statuses (Rouse & Austin, 2002). However, these studies have primarily concentrated on intrinsic values. Adolescents with high self-transcendence values, which prioritize helping others and contributing to society (Johnson, 2001; Johnson & Elder, 2002), often develop a deeper understanding of life and learning (Yeager & Bundick, 2009), potentially enhancing their engagement in education (Otis et al., 2005). However, there is a lack of empirical evidence examining the relationship between self-transcendence values and school engagement, as well as the relative strength of the correlation between different values and school engagement. Additionally, research on the mediating mechanisms linking different values to school engagement is scarce.

### 1.2. Achievement Goals and School Engagement

Achievement goals play a pivotal role in school engagement. These goals represent the competence-related outcomes in achievement contexts that individuals either aim for or strive to avoid (Elliot, 2005; Hulleman et al., 2010). Broadly, achievement goals can be categorized into four types, namely mastery approach, mastery avoidance, performance approach, and performance avoidance (Elliot & McGregor, 2001; Elliot & Thrash, 2001). Mastery approach goals focus on the pursuit of personal growth, competency development, and reaching one’s fullest potential (Elliot & McGregor, 2001; Grant & Dweck, 2003; Hulleman et al., 2010). Conversely, individuals with mastery avoidance orientations aim to steer clear of misunderstandings, prioritizing this over learning or mastering a task. Performance approach goals, on the other hand, center on showcasing competence to others, while performance avoidance goals arise from a wish to prevent appearing inept in front of others.

Research has consistently shown that mastery approach goals have a positive association with cognitive, behavioral, and emotional engagement (Cho et al., 2019; Ferrell, 2012; Lau et al., 2008; Q. Liu et al., 2023; Luo & Luo, 2022; Mih et al., 2015). Additionally, studies have highlighted positive correlations between performance approach goals with both cognitive and emotional engagement (Lau et al., 2008; Tas, 2016). However, both performance avoidance and mastery avoidance goals have been found to have a negative correlation with school engagement (Cho et al., 2019; Elliot & McGregor, 2001; Lau et al., 2008).

### 1.3. Personal Values and Achievement Goals

Adolescents’ achievement goals are shaped by their personal values. Yeager and Bundick (2009) postulated a connection between personal values and achievement goals. They believed that adolescents holding intrinsic and self-transcendence values would lean towards mastery goals. In contrast, those with extrinsic values might favor performance goals. However, this theory was not empirically tested in their study. Ku et al. (2014) conducted empirical research to assess how materialistic values affect achievement goals. Their findings indicated that materialistic values were negatively correlated with mastery goals and positively correlated with performance avoidance goals. Surprisingly, no relation was found with performance approach goals.

Notably, these studies did not differentiate between the subtypes of mastery goals, namely mastery approach and mastery avoidance, which are recognized as representing distinct learning motivations (Elliot, 1999; Pintrich, 2000). Based on previous research, self-improvement and self-transcendence values may be strongly linked to mastery approach goals, while materialistic values may correlate with mastery avoidance goals. Specifically, self-improvement values encourage adolescents to develop intrinsic learning motivation, inspiring them to diligently acquire and master knowledge (Schwartz, 1992). Similarly, self-transcendence values motivate students to gain knowledge and skills that contribute to society (Kasser & Ryan, 1996). Thus, both types of values are likely positively associated with mastery approach goals. Adolescents with high materialistic values often view success as achieving external milestones. Consequently, their fear of failing to meet social or economic expectations may lead them to shy away from academic challenges, predisposing them towards mastery avoidance goals (Elliot & Thrash, 2001). However, these relationships still require empirical verification. Furthermore, it remains to be examined whether achievement goals mediate the relationship between personal values and school engagement.

The future-oriented motivation and self-regulation model proposed by Miller and Brickman (2004) can be used to hypothesize the relationship between personal values, achievement goals, and school engagement. This model is particularly relevant to our research question because it may provide a clearer explanation of the relationships between these elements than other models. It suggests that adolescents’ personal values shape their task values and proximal goals, which in turn influence their engagement with tasks. Achievement goals, which are part of these task values and goals, may be influenced by personal values and potentially impact school engagement. Thus, the model suggests that achievement goals might mediate the relationship between personal values and school engagement. This study investigated whether different types of achievement goals served as mediators between various personal values and school engagement, thereby expanding the model. This study also held important practical significance, as the results offered guidance for the direction of personal value education.

### 1.4. Cultural Background Differences

Most of the studies examining the relationships between personal values and associated outcomes have been conducted in Western countries. As China is a developing country, it is essential to explore how different personal values affect the academic performance among Chinese adolescents.

Although research shows cross-cultural consistency in the influence of materialistic values on achievement goals among students in Britain and China (Ku et al., 2014), it is important to note that the Chinese participants in this study were from Hong Kong. There are significant differences in economic background, educational concepts, and social expectations between Hong Kong and many inland cities in China. In these less economically developed inland regions, good grades and higher education are often viewed as essential for improving both individual and family economic conditions. Consequently, materialistic values may prompt teenagers in these areas to pursue performance approach goals to meet material and social expectations.

Given the emphasis on collectivism in Chinese society, teenagers with higher levels of self-transcendence values may be more engaged in learning. Therefore, it also remains to be explored whether the positive correlation between self-transcendence values and school engagement is stronger than the correlation between self-improvement values and school engagement.

In addition, in the context of Chinese culture, the relationship between performance approach goals and school engagement, as well as between mastery avoidance goals and school engagement, may differ from findings in Western studies. Research in Western countries has identified a significant positive correlation between performance approach goals and school engagement (Lau et al., 2008; Tas, 2016) and a significant negative correlation between mastery avoidance goals and school engagement (Cho et al., 2019; Elliot & McGregor, 2001; Lau et al., 2008). However, in China, families and society often hold high expectations for adolescents’ academic achievements. Many teenagers see achieving good grades as a necessary task to fulfill others’ expectations, which may not enhance their engagement in school. It can be seen that performance approach goals may not necessarily motivate Chinese teenagers to commit to learning. Additionally, teenagers who fear not fully mastering the material may still participate in learning to prepare for exams, suggesting that mastery avoidance goals do not always deter engagement in learning.

In summary, it is essential to choose adolescents from China, particularly those from inland cities, as participants to study the relationships among various personal values, achievement goals, and school engagement.

### 1.5. Present Study

This study sought to examine the effect of various personal values on school engagement in Chinese adolescents, as well as the role of four achievement goals as potential mediators. This study focused on three types of personal values, namely materialistic values, self-improvement values, and self-transcendence values. These values align with the three components of happiness proposed by Seligman. They have been identified as common personal values within the Chinese cultural context (Xiang, 2014) and have been shown to relate to the learning experiences of adolescents and college students (Ku et al., 2014; Roeser et al., 2002; Rouse & Austin, 2002; Yeager & Bundick, 2009). In contrast, other values, such as tradition, have not been demonstrated to be linked to academic performance and therefore were not included in this study. According to the definitions provided, materialistic values correspond to the extrinsic values mentioned earlier and self-improvement values correspond to intrinsic values. The findings of present study can inform the development of value education programs for Chinese adolescents.

Drawing from previous research, we formulated several hypotheses for our investigation. First, we hypothesized that the materialistic values of Chinese adolescents would positively correlate with both their performance approach and mastery avoidance goals, although these achievement goals might be not further associated with their school engagement. We explored the relationships between both self-improvement and self-transcendence values and these achievement goals as an initial inquiry. Second, we anticipated that both self-improvement and self-transcendence values would positively correlate with school engagement, potentially mediated by an increase in mastery approach goals. We examined whether mastery approach goals mediated the relationship between materialistic values and school engagement as an exploratory work. Third, we expected higher levels of materialistic values to correlate with an increased tendency towards performance avoidance goals, which would be in turn negatively related to school engagement. We also explored whether performance avoidance goals mediated the relationship between self-improvement and self-transcendence values and school engagement. Fourth, we anticipated that the positive correlation between self-transcendence values and school engagement would be stronger than that between self-improvement values and school engagement. Finally, we conducted an exploratory analysis of the pairwise correlations among the three types of personal values.

## 2. Materials and Methods

### 2.1. Participants and Procedure

Participants were senior high school students from a public high school in Changchun, Jilin province, China. School principals and teachers granted permission for classes to take part in this research. All participants completed the questionnaire within one week, consistent with the study’s cross-sectional design. Of the initial 750 students approached, 93.33% agreed to take part, completing an anonymous online questionnaire in the school’s computer room. The final participant count stood at 700 students (55.57% male and 44.43% female). On average, participants were 16.91 years old with a standard deviation of 0.43 years in age. The study was approved by the School of Psychology’s Ethics Review and Research Committee. Both students and their parents provided informed consent. Students were guaranteed confidentiality, and their information would only be used for research purposes.

### 2.2. Measures

#### 2.2.1. Personal Values Scale

The Personal Values Scale (PVS) (Xiang, 2014) measured three distinct personal values, namely materialistic, self-improvement, and self-transcendence values. Materialistic values underscore the importance of possessions, self-improvement values stress the significance of personal growth, and self-transcendence values highlight altruism. This scale included 17 items, 7 pertaining to materialistic values (e.g., ‘I value associations with economically strong individuals’), 5 for self-improvement values (e.g., ‘I am open to confronting challenges’), and 5 for self-transcendence values (e.g., ‘I engage in social welfare activities in my free time’). Responses were gauged on a five-point Likert scale, ranging from 1 (‘strongly disagree’) to 5 (‘strongly agree’). For this study, subscale scores for each participant were derived by averaging the respective item scores. The scales demonstrated solid internal consistency and construct validity. Cronbach’s α values for the materialistic, self-improvement, and self-transcendence subscales were 0.80, 0.75, and 0.66, respectively. The confirmatory factor analysis (CFA) revealed an optimal fit of χ^2^/df = 2.64. The goodness-of-fit index (GFI), incremental fit index (IFI), Tucker–Lewis index (TLI), and comparative fit index (CFI) were 0.95, 0.93, 0.91, and 0.93, respectively. Additionally, the root mean square error of approximation (RMSEA) and standardized root mean square residual (SRMR) both stood at 0.05, further affirming the instrument’s construct validity.

#### 2.2.2. Achievement Goal Orientation Scale

The Achievement Goal Orientation Scale (H. Liu & Guo, 2003) assessed four distinct achievement goals, which were mastery approach, mastery avoidance, performance approach, and performance avoidance goals. Mastery approach goals emphasize learning, understanding, and mastering tasks. Mastery avoidance goals target avoiding misunderstandings. Performance approach goals aim to outshine peers, while performance avoidance goals strive to avoid appearing inferior. This scale featured 29 items, 9 for mastery approach goals (e.g., ‘I enjoy learning to expand my knowledge’), 5 for mastery avoidance goals (e.g., ‘I often worry I may not fully grasp classroom teachings’), 9 for performance approach goals (e.g., ‘I study diligently to improve my class rank’), and 6 for performance avoidance goals (e.g., ‘I hesitate to speak up in class to avoid looking foolish’). Responses utilized a five-point Likert scale, ranging from 1 (‘strongly disagree’) to 5 (‘strongly agree’). Subscale scores were derived by averaging related items. The study reported robust construct validity and internal consistency. The model fit was characterized by χ^2^/df = 2.75. The GFI, IFI, and CFI were 0.91, 0.90, and 0.90, respectively, while the RMSEA stood at 0.05 and the SRMR at 0.07. Cronbach’s α values for the subscales of mastery approach, mastery avoidance, performance approach, and performance avoidance goals were 0.79, 0.75, 0.83, and 0.80, respectively.

#### 2.2.3. School Engagement Scale

School engagement encompasses cognitive, emotional, and behavioral aspects. These were assessed through a questionnaire featuring 3 items on behavioral engagement (e.g., ‘I would skip school for an entire day’), 5 items on emotional engagement (e.g., ‘I dislike learning’), and 5 items on cognitive engagement (e.g., ‘I can achieve good grades’) (Zhang, 2013). Responses were recorded on a four-point Likert scale. For behavioral and emotional engagement, scores ranged from 1 (‘strongly agree’) to 4 (‘strongly disagree’). In contrast, cognitive engagement scores varied from 1 (‘strongly disagree’) to 4 (‘strongly agree’). To more directly reflect school engagement, we calculated an overall score for each participant by averaging their responses to all items. The instrument demonstrated sound construct validity and internal consistency. The confirmatory factor analysis (CFA) model showed a good fit with χ^2^/df = 2.90. The GFI, IFI, TLI, and CFI were 0.96, 0.93, 0.91, and 0.93, respectively. Additionally, the RMSEA stood at 0.05, and the SRMR was 0.05. The overall Cronbach’s α for the questionnaire was 0.71.

### 2.3. Data Analysis

Potential issues of common method bias were addressed, given that data came from multiple scales. To evaluate this, we employed Harman’s single-factor test. Subsequently, we presented descriptive statistics and correlations. We then employed regression analyses to examine the effects of different personal values on school engagement. Lastly, we utilized AMOS 21.0 to construct a structural equation model, determining if the four achievement goals served as mediators between personal values and school engagement.

## 3. Results

### 3.1. Common Method Bias Test

Method bias constitutes a primary source of measurement error. Specifically, common method bias can introduce systematic measurement errors that might severely skew research findings (Podsakoff et al., 2003). To address potential biases in this study, we employed Harman’s single-factor test. By loading all items of the variables into an exploratory factor analysis (Andersson & Bateman, 1997), we found that the principal factor accounted for only 13.02% of the total variance. As this is less than 40%, it suggests that common method bias was not significant in our research.

### 3.2. Descriptive Statistics and Correlations

Table 1 displays the means and standard deviations for all variables, as well as the correlation coefficients between them.

The correlation analysis revealed several relationships among the variables. Self-transcendence values had a negative correlation with materialistic values (*r* = −0.10, *p* < 0.01) but were positively associated with self-improvement values (*r* = 0.41, *p* < 0.001). Both self-transcendence and self-improvement values showed positive correlations with mastery approach goals (*r*_1_ = 0.29, *p* < 0.001; *r*_2_ = 0.50, *p* < 0.001), performance approach goals (*r*_1_ = 0.08, *p* < 0.05; *r*_2_ = 0.13, *p* < 0.001), and school engagement (*r*_1_ = 0.28, *p* < 0.001; *r*_2_ = 0.44, *p* < 0.001). Conversely, they were negatively associated with performance avoidance goals (*r*_1_ = −0.10, *p* < 0.05; *r*_2_ = −0.23, *p* < 0.001). Materialistic values demonstrated positive correlations with performance approach goals (*r* = 0.28, *p* < 0.001), performance avoidance goals (*r* = 0.11, *p* < 0.01), and mastery avoidance goals (*r* = 0.11, *p* < 0.01) but were negatively associated with school engagement (*r* = −0.11, *p* < 0.01).

Given the significant associations between personal values, achievement goals, and school engagement, further statistical analyses are warranted.

### 3.3. Influence of Personal Values on School Engagement

Prior to the mediation analysis, we conducted regressive analyses with school engagement as the dependent variable and each of the three types of personal values as the independent variables. The outcomes are presented in Table 2.

As depicted in Table 2, both self-improvement and self-transcendence values positively influenced school engagement (β_1_ = 0.44, *p* < 0.001; β_2_ = 0.28, *p* < 0.001). In contrast, materialistic values had a negative impact on school engagement (β = −0.11, *p* < 0.01).

### 3.4. Mediating Role of Achievement Goals

**Figure 1:**
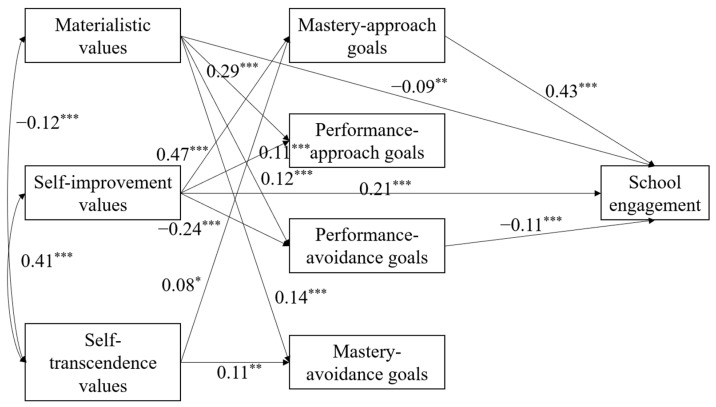
Achievement goals bridging the link between values and school engagement. Note: standardized coefficients of significant paths are reported; * *p* < 0.05, ** *p* < 0.01, *** *p* < 0.001.

We employed a structural equation model (SEM) to investigate the potential mediating effect of achievement goals between personal values and school engagement, as illustrated in Figure 1. The model fit was outstanding, at χ^2^/df = 1.54. The values for the GFI, NFI, RFI, IFI, TLI, and CFI were 0.996, 0.988, 0.959, 0.996, 0.985, and 0.996, respectively. Furthermore, the RMSEA stood at 0.03, while the SRMR was 0.02.

Figure 1 illustrates several mediation pathways. Stronger materialistic values were linked to more performance avoidance goals (β = 0.12, *p* < 0.001), which in turn were associated with decreased school engagement (β = −0.11, *p* < 0.001). In this study, we used the ratio of mediation effect to total effect as the effect size metric for mediation (Fang et al., 2012). Consequently, the effect size for the mediating role of performance avoidance goals between materialistic values and school engagement was 0.13. Higher levels of self-improvement values were associated with more mastery approach goals (β = 0.47, *p* < 0.001), which were further linked to increased school engagement (β = 0.43, *p* < 0.001). The effect size for the mediation by mastery approach goals was 0.46. An interesting secondary effect was observed where self-improvement values, positively associated with school engagement, were also mediated by fewer performance avoidance goals (β_1_ = −0.24, *p* < 0.001; β_2_ = −0.11, *p* < 0.001), with an effect size of 0.06. Self-transcendence values were positively associated with mastery approach goals (β = 0.08, *p* < 0.05), which were then related to enhanced school engagement (β = 0.43, *p* < 0.001). However, due to the insignificant direct correlation between self-transcendence values and school engagement and the absence of other mediating variables, the effect size for the mediation by mastery approach goals was not estimated.

Both self-improvement values and self-transcendence values were significantly positively correlated with school engagement through achievement goals. Therefore, it is important to compare the effects of self-transcendence values on school engagement with those of self-improvement values. To compare the relative magnitude of their indirect effects on school engagement, we examined the 95% confidence intervals (CIs) of these effects. If the CIs of the indirect effects of self-improvement and self-transcendence values on school engagement do not overlap, it indicates a significant difference in the magnitude of the effects. The results showed that the 95% CI for the indirect effects of self-improvement values on school engagement was [0.18, 0.28], and for the indirect effects of self-transcendence values on school engagement, it was [0.01, 0.07]. Similarly, the 95% CIs for the total effects of self-improvement and self-transcendence values on school engagement were [0.36, 0.50] and [0.01, 0.07], respectively. These findings suggest that both the indirect and total effects of self-improvement values on school engagement were significantly greater than those of self-transcendence values. This suggests that for senior high school students, self-improvement values may be more crucial than self-transcendence values in promoting good academic performance.

## 4. Discussion

Numerous studies in the realm of personal values have focused on how these values impact the career development of college students and employees (Añaña & Nique, 2010; Fearon et al., 2018; Francescato et al., 2020; Gaile et al., 2022; Sagiv et al., 2017; Sato et al., 2021; Schwartz & Bardi, 2001). However, personal values typically form during high school (Johnson, 2001; Johnson & Elder, 2002). At this stage, students prioritize academic performance over immediate career decisions. While some research has delved into the relationship between personal values and academic performance (Gamage et al., 2021; Parks-Leduc et al., 2022; Roeser et al., 2002; Rouse & Austin, 2002), this area warrants further exploration. This study aims to delve deeper into how various personal values influence high school students’ school engagement and to uncover the mediating mechanisms.

The results suggested that materialistic values have a nuanced association with academic performance, encompassing both potential benefits and drawbacks. Our study suggests a positive correlation between materialistic values and performance approach goals, deviating from Ku et al. (2014), who observed no such relationship. This difference might be attributed to varying cultural backgrounds. Participants in Ku et al.’s study were from economically affluent regions, where good grades are not necessarily crucial for achieving economic success. Consequently, the lack of a significant correlation between materialistic values and performance approach goals in their study is understandable. In contrast, our study involved participants from less developed areas, where good grades and higher education are considered essential for economic improvement. Therefore, the materialistic values of participants from these areas likely motivate them to pursue performance approach goals to meet material and social expectations. These goals can benefit adolescents’ learning enthusiasm to a certain extent.

However, it is essential to recognize the potential pitfalls of materialistic values in academic settings. While these values may be positively related to performance approach goals, Elliot and Harackiewicz (1996) note that these goals can also induce detrimental emotions like anxiety. Such emotions could hinder the implementation of effective learning strategies. Furthermore, our study found a positive correlation between materialistic values and mastery avoidance goals. This may be because adolescents with higher levels of materialistic values often view success in terms of external achievements, leading them to fear failure. This fear may drive them to avoid academic challenges that could result in failure, making them more inclined to adopt mastery avoidance goals (Elliot & Thrash, 2001). This finding supports our research hypothesis. However, no significant correlation was found between materialistic values and mastery approach goals, which emphasize personal growth and competency development (Elliot & McGregor, 2001; Grant & Dweck, 2003; Hulleman et al., 2010). Since adolescents with higher levels of materialistic values prioritize external rewards over personal interests and self-growth (Kasser & Ryan, 1996), materialistic values may not be effectively associated with mastery approach goals. These findings suggest that the relationship between materialistic values and mastery approach goals differs from that with mastery avoidance goals. The results indicate that adolescents who pursue external rewards may be more concerned with performing well in front of others and avoiding partial understanding of knowledge. Although materialistic values may be beneficial for adolescents’ learning enthusiasm to some extent, they do not consistently motivate the pursuit of personal growth and skill development. On the contrary, these values may negatively affect learning by encouraging a focus on avoiding incomplete mastery of knowledge rather than on fully mastering it (Elliot, 1999; Pintrich, 2000).

The results of the mediation test further highlighted the potential drawbacks of materialistic values in academic settings. Materialistic values were found to be negatively related to school engagement through performance avoidance goals. Specifically, adolescents with higher materialistic values tended to have more performance avoidance goals, which were then negatively associated with their school engagement. A possible explanation is that compared to adolescents with lower levels of materialistic values, those with higher levels may have greater concerns about appearing unskilled or inadequate in academic settings (Ku et al., 2014). Such apprehensions could deter them from fully engaging in the learning process (Elliot & Harackiewicz, 1996). Alternatively, it might be suggested that students with stronger materialistic inclinations, compared to those with weaker ones, worry more about not achieving stellar grades or securing a spot in a prestigious university. This anxiety, rooted in fears of not fulfilling their aspirations for wealth and status, could fuel a heightened avoidance of underperformance, further curtailing their engagement in school.

Adolescents’ materialistic values were also found to be directly and negatively correlated with their school engagement, although this correlation was weakly significant. Nevertheless, the significance of this weak correlation warrants attention. A plausible explanation is that the collectivist culture in China may diminish the negative correlation between materialistic values and school engagement. In a collectivist culture, students’ academic achievements often reflect not only their personal honor but also the honor of their families. Thus, even if teenagers possess high levels of materialistic values, social norms and collective expectations might mitigate the adverse effects of materialistic values on school engagement to some extent. However, it is important to note that the negative effects had not been completely eliminated.

The findings of this study highlighted that both self-improvement and self-transcendence values were positively associated with academic performance. Specifically, self-improvement values were positively linked to mastery approach goals, which in turn were positively related to school engagement. One possible explanation is that these values help adolescents connect their current learning with future career aspirations, enabling them to understand the deeper significance of life and learning (Yeager & Bundick, 2009). This orientation fosters a focus on mastering tasks, which ultimately enhances school engagement (Otis et al., 2005). Additionally, self-improvement values were positively correlated with school engagement, with this relationship partially mediated by fewer performance avoidance goals. A possible reason for this is that adolescents with higher self-improvement values prioritize developing their abilities. This focus on personal growth may reduce their fear of appearing incompetent, leading to greater school engagement.

Similarly, self-transcendence values were positively linked to mastery approach goals, which in turn were positively related to school engagement. However, it is important to note that the correlation between self-transcendence values and mastery approach goals was only weakly significant according to the correlation coefficients. Additionally, there was no significant direct correlation between self-transcendence values and school engagement. These findings suggest that in collectivist cultures, while self-transcendence values of senior high school students are positively associated with academic performance, this association is relatively weak. This might be because in China, the primary objective for senior high school students is often gaining admission to a prestigious university. Altruistic goals, such as helping others and contributing to society, may be seen as secondary. Therefore, despite the influence of collectivist culture, the impact of self-transcendence values on academic performance is limited because societal contribution is a secondary goal for these students.

We further compared the overall strength of the correlation between self-improvement values and school engagement to that between self-transcendence values and school engagement. The result indicated a stronger relationship with school engagement for self-improvement values than for self-transcendence values. Contrary to our hypothesis, this suggests that in a collectivist culture, the correlation between self-transcendence values and academic performance is not stronger. This finding further supports the explanation that although senior high school students are influenced by collectivist culture, their primary goal is self-improvement to gain admission to a prestigious university rather than to serve society. Therefore, self-improvement values may be more pivotal in motivating students to engage deeply in their studies.

An interesting finding of this study was the significant positive correlation between self-transcendence values and mastery avoidance goals. One possible explanation is that teenagers who prioritize serving others may feel the need to acquire specific knowledge and skills to do so effectively. As a result, they may set higher standards for understanding to avoid incomplete comprehension. However, the relationship between self-transcendence values and school engagement was not mediated by either performance avoidance or performance approach goals. This finding extends previous research, suggesting that adolescents who value serving others are less focused on their performance in front of others.

The results of our study also showed no significant correlation between performance approach goals and school engagement nor between mastery avoidance goals and school engagement. These findings differ from previous research (Cho et al., 2019; Elliot & McGregor, 2001; Lau et al., 2008; Tas, 2016) and support our hypotheses. Cultural differences may explain these discrepancies. In Chinese culture, families and society often place high expectations on adolescents’ academic achievement. For many teenagers, academic achievement is seen as a task to meet others’ expectations rather than a personal goal. As a result, it may not lead to increased school engagement. Furthermore, in Chinese culture, teenagers who fear not fully mastering knowledge may continue participating in learning, primarily to prepare for exams. Therefore, it is reasonable that no significant correlation was found between performance approach goals and school engagement nor between mastery avoidance goals and school engagement.

It is important to note that the three types of personal values investigated in this study were interrelated rather than existing in isolation. Specifically, the result suggested that adolescents with pronounced self-transcendence values often exhibited elevated self-improvement values, aligning with previous research (Tanaka et al., 2009). Additionally, higher self-transcendence values were associated with lower materialistic values. This finding extends previous research, suggesting that individuals who prioritize serving others are less likely to pursue wealth or status. No significant correlation was found between self-improvement values and materialistic values. This finding aligns with prior research (Tessier et al., 2021). This suggests that adolescents’ pursuit of personal growth does not necessarily conflict with their desire for money or status.

Given these interrelationships, fostering self-transcendence values in adolescents might be a strategic approach to enhancing their academic development. This is because self-transcendence values not only correlate positively with self-improvement values but also negatively with materialistic values. However, it is impractical to expect every individual to develop high levels of self-transcendence values to become a ‘contributor’. Moreover, self-improvement values have been shown to be more strongly linked to academic performance than self-transcendence values. Therefore, while cultivating self-transcendence values is beneficial, the primary focus of personal value education should remain on fostering self-improvement values.

To cultivate adolescents’ self-improvement and self-transcendence values, educators can employ strategies such as role-model learning. This approach involves encouraging individuals with high levels of self-improvement and self-transcendence values to share their stories, helping adolescents understand the joy of learning and serving others. Educators should tailor this approach by choosing role models who reflect the specific economic and social challenges of their area. For example, in economically disadvantaged rural areas, role models might be individuals who have advanced through local educational opportunities or who have improved local infrastructure. In contrast, in affluent urban areas, role models could be successful entrepreneurs or professionals. Additionally, educators can engage students in scientific experiments and volunteer services tailored to their environments. For instance, in rural areas, students could participate in agricultural experiments that focus on conservation techniques beneficial to their local environment, enhancing both scientific literacy and community stewardship. In urban settings, opportunities might include volunteering at nursing homes, fostering a sense of service among students. These activities not only expand students’ horizons but also motivate them towards personal and community growth, leading to greater learning engagement (Damon, 2009; Deci & Ryan, 2000).

### Limitations and Future Directions

While this study offers meaningful results, certain limitations warrant consideration in future research. Firstly, relying solely on self-report questionnaires introduces certain limitations, such as common method bias and social desirability bias. Although the common method bias test suggested minimal systematic measurement errors in this study, future research should include more objective measures, such as actual academic performance records, to mitigate other potential biases like social desirability. Secondly, our research used a cross-sectional design, which limits our ability to draw causal conclusions. Future studies would benefit from more robust designs, such as longitudinal or cross-lagged analyses, to further corroborate these findings and establish causality. Thirdly, the participants in this study were from a single region, limiting the generalizability of the results. Economic development levels may impact these findings. For example, our study identified a positive correlation between materialistic values and performance approach goals in adolescents from less developed areas in China, whereas Ku et al. (2014) did not observe such a relationship in adolescents from economically affluent regions of China. Therefore, future research should include participants from regions with varying economic development levels within China to explore the similarities and differences in findings. Fourthly, the relationship between other types of personal values, such as tradition and universalism, and academic performance remains unexplored. Future research should examine these relationships to provide more comprehensive guidance for value education.

## 5. Conclusions

Our findings indicate that achievement goal orientations mediate the relationship between personal values and school engagement. Specifically, materialistic values were positively correlated with performance avoidance goals, which in turn were associated with decreased school engagement. In contrast, self-improvement values were positively associated with school engagement, and this connection was mediated by more mastery approach goals and fewer performance avoidance goals. Similarly, self-transcendence values were positively related to mastery approach goals, which were subsequently associated with increased school engagement. Additionally, although both self-improvement and self-transcendence values positively correlated with adolescents’ school engagement, the correlation was stronger for self-improvement values. This study demonstrates that promoting self-improvement and self-transcendence values, especially self-improvement values, fosters positive achievement goal orientations like mastery approach goals, which in turn enhance school engagement.

## Figures and Tables

**Table 1:** Descriptive statistics and correlations of variables (total *n* = 700).

Variables	M	SD	1	2	3	4	5	6	7	8
1. Materialistic values	3.05	0.77	1							
2. Self-improvement values	3.91	0.67	0.04	1						
3. Self-transcendence values	3.45	0.70	−0.10 **	0.41 ***	1					
4. Mastery approach goals	3.32	0.59	−0.04	0.50 ***	0.29 ***	1				
5. Performance approach goals	3.33	0.69	0.28 ***	0.13 ***	0.08 *	0.27 ***	1			
6. Performance avoidance goals	2.03	0.70	0.11 **	−0.23 ***	−0.10 *	−0.11 **	0.26 ***	1		
7. Mastery avoidance goals	3.59	0.75	0.11 **	0.06	0.11 **	0.27 ***	0.35 ***	0.25 ***	1	
8. School engagement	3.06	0.37	−0.11 **	0.44 ***	0.28 ***	0.55 ***	0.07 *	−0.22 ***	0.07	1

Note: * *p* < 0.05, ** *p* < 0.01, *** *p* < 0.001.

**Table 2:** Impact of personal values on school engagement.

X	β	t	F	R^2^
Self-improvement values	0.44	13.05	170.19 ***	0.20
Self-transcendence values	0.28	7.67	58.76 ***	0.08
Materialistic values	−0.11	−2.92	8.52 **	0.01

Note: ** *p* < 0.01, *** *p* < 0.001.

## Data Availability

The data that support the findings of this study are available from the corresponding author upon reasonable request.
